# Filament-Producing Mutants of Influenza A/Puerto Rico/8/1934 (H1N1) Virus Have Higher Neuraminidase Activities than the Spherical Wild-Type

**DOI:** 10.1371/journal.pone.0112462

**Published:** 2014-11-10

**Authors:** Jill Seladi-Schulman, Patricia J. Campbell, Suganthi Suppiah, John Steel, Anice C. Lowen

**Affiliations:** Department of Microbiology and Immunology, Emory University School of Medicine, Atlanta, Georgia, United States of America; University of Georgia, United States of America

## Abstract

Influenza virus exhibits two morphologies – spherical and filamentous. Strains that have been grown extensively in laboratory substrates are comprised predominantly of spherical virions while clinical or low passage isolates produce a mixture of spheres and filamentous virions of varying lengths. The filamentous morphology can be lost upon continued passage in embryonated chicken eggs, a common laboratory substrate for influenza viruses. The fact that the filamentous morphology is maintained in nature but lost in favor of a spherical morphology *in ovo* suggests that filaments confer a selective advantage within the infected host that is not necessary for growth in laboratory substrates. Indeed, we have recently shown that filament-producing variant viruses are selected upon passage of the spherical laboratory strain A/Puerto Rico/8/1934 (H1N1) [PR8] in guinea pigs. Toward determining the nature of the selective advantage conferred by filaments, we sought to identify functional differences between spherical and filamentous particles. We compared the wild-type PR8 virus to two previously characterized recombinant PR8 viruses in which single point mutations within M1 confer a filamentous morphology. Our results indicate that these filamentous PR8 mutants have higher neuraminidase activities than the spherical PR8 virus. Conversely, no differences were observed in HAU:PFU or HAU:RNA ratios, binding avidity, sensitivity to immune serum in hemagglutination inhibition assays, or virion stability at elevated temperatures. Based on these results, we propose that the pleomorphic nature of influenza virus particles is important for the optimization of neuraminidase functions *in vivo*.

## Introduction

Influenza A virus (IAV) is an enveloped virus containing eight negative-sense RNA gene segments [1]. It is the causative agent of seasonal epidemics of respiratory illness as well as occasional pandemics, the most recent of which occurred in 2009 [2]. IAV is pleomorphic, producing virions of spherical and filamentous morphology [3]. Strains that produce predominantly spherical or ovoid virions have typically been passaged many times within laboratory substrates, while filament-producing strains occur in primary or low passage isolates [4], [5]. Filaments are of variable length and can be up to 30 µm long [6]. Herein, we define filaments as any virion 300 nm in length or longer (≥3x the diameter of a typical spherical virion). Studies performed using reverse genetics systems have identified the M1 matrix protein as the major genetic determinant of virion morphology, however portions of the viral nucleoprotein (NP) as well as the cytoplasmic tails of the M2 ion channel, hemagglutinin (HA) and neuraminidase (NA) proteins have been shown to affect virion morphology as well [7]–[13].

Early observations showed that the filamentous morphology is gradually lost upon continued passage in embryonated chicken eggs in favor of a more spherical morphology [14], [15]. We also observed that filaments could be lost following ten passages in eggs or MDCK cells, but that this phenotypic change was not required for robust adaptation to either substrate [5]. The fact that filaments are maintained in nature while dispensable for growth in laboratory substrates suggests that the filamentous morphology provides a selective advantage within the infected host that is not necessary for growth in the laboratory. Previously, we showed that passaging of the spherical laboratory strain A/Puerto Rico/8/1934 (H1N1) [PR8] twelve times in guinea pigs led to the emergence of virions that are filamentous in morphology [5]. Through sequencing of the M1 matrix gene of the passage 12 (P12) virus pool, we identified several coding mutations within M1. Individual introduction of four of these amino acid changes using reverse genetics yielded mutant viruses that produced significantly more filaments than the wild-type [p<0.05; difference in proportions test] [5].

While selection for a filamentous morphology through passaging in an animal host confirms an advantage of filamentous virions *in vivo*, the nature of the selective advantage remains unclear. To determine the advantage filament-producing viruses have over their spherical counterparts, we used recombinant wild-type PR8 (rPR8wt) and two previously characterized filamentous M1 mutants – rPR8 M1 N87S (N87S) and rPR8 M1 R101G (R101G) [5]. Our published particle counts showed that 16% and 41% of virions were filamentous for N87S and R101G viruses, respectively, compared to 4% for PR8wt [5]. These three viruses present an ideal system in which to address the differences between exclusively spherical and filament-producing IAV, for the following reasons: i) the mutant strains are highly similar genetically to the rPR8wt, simplifying the interpretation of results; ii) the mutations used arose naturally, minimizing the likelihood of disrupting viral functions through their introduction; and iii) the mutant viruses differ significantly from rPR8wt in terms of filament production. Thus, rPR8wt, N87S, and R101G viruses were analyzed in a series of *in vitro* assays. We hypothesized that functional differences between spherical and filamentous virions might arise due to their differing surface areas, and therefore focused our efforts on the two surface glycoproteins of IAV, HA and NA. We tested whether the HA avidity or NA activity per virion differed between the spherical rPR8wt virus and filament producing strains. In addition, the ratio of hemagglutination units (HAU) to plaque forming units (PFU), the ratio of HAU to RNA copies, and virion stability at elevated temperatures were investigated. Our findings suggest a role of the viral NA protein in the fitness advantage conferred by filamentous virion morphology: by two independent measures, the two filamentous rPR8 mutants displayed higher neuraminidase activities compared to rPR8wt, but did not differ significantly in binding avidity, inhibition of binding by antiserum, infectivity or thermostability.

## Material and Methods

### Ethics statement

This study was performed in accordance with the recommendations in the Guide for the Care and Use of Laboratory Animals of the National Institutes of Health. Animal husbandry and experimental procedures were approved by the Emory University Institutional Animal Care and Use Committee (IACUC protocol #2000719).

### Viruses and cells

The rPR8wt, rPR8 M1 N87S, and rPR8 M1 R101G viruses were generated using reverse genetics as previously described [16]–[18]. Briefly, rPR8-based viruses were recovered following eight (pDZ) plasmid transfection of 293T cells and subsequent inoculation of transfected cells and culture medium into 9–11 day old embryonated chicken’s eggs. Stocks of the rPR8 wild-type virus and mutants were generated in 9–11 day old embryonated chicken’s eggs. Influenza A/Udorn/301/1972 (H3N2) virus was grown in MDCK cells. Influenza A/Anhui/1/2013 (H7N9) virus was grown in eggs under enhanced BSL3 containment and inactivated by addition of beta-propiolactone (BPL) prior to removal from the BSL3 facility.

Washed chicken red blood cells from Lampire Biological were used for all hemagglutination-based assays. MDCK cells, a kind gift of Peter Palese, were maintained in minimal essential medium supplemented with 10% fetal bovine serum and penicillin/streptomycin. 293T cells (ATCC), used for virus rescue by reverse genetics, were maintained in Dulbecco’s minimal essential medium supplemented with 10% fetal bovine serum. Embryonated chickens’ eggs were obtained from Hy-Line International and incubated at 37°C, with rocking, for 9–11 days prior to inoculation with IAV.

### Infectious titer comparison

Viruses were diluted to concentration of 128 HAU. Hemagglutination units, or HAU, are defined as the reciprocal of the highest dilution of virus still allowing agglutination of red blood cells. After confirming the HA titer, viruses were titrated in triplicate by plaque assay of 10-fold serial dilutions on MDCK cells. A Student *t*-test was used to compare the infectious titers of each mutant virus to rPR8wt virus.

### C_q_ value comparison

Viruses were diluted to concentration of 128 HAU. After confirming the HA titer, RNA was extracted from 160 µl of each diluted virus sample using the QIAamp Viral RNA Mini Kit (QIAGEN), according to the manufacturer’s instructions. cDNA was generated using Maxima reverse transcriptase (Thermo Scientific) and a universal forward primer (GGCCAGCAAAAGCAGG). Quantitative PCR was then performed on a Bio-Rad CFX384 thermocycler using the cDNA as template, SsoFast EvaGreen Supermix (Bio-Rad), and primers specific for the NP segment (F: TATTCGTCTCAGGGAGCAAAAGCAGG [19] and R: CTGATTTCAGTGGCATTCTGGC). Each cDNA was analyzed in triplicate and the resulting cycle threshold (C_q_) values were recorded. Average C_q_ values shown in Table 1 were calculated by first converting each C_q_ value to 2^(−Cq)^, calculating the arithmetic mean, and then taking the –log_2_ of the arithmetic mean.

**Table 1:** 128 HAU of rPR8wt, rPR8 M1 N87S and rPR8 M1 R101G viruses comprise comparable infectious titers and genome copies.

Virus	Infectious titer (PFU/ml)^a^	Average infectious titer (PFU/ml)	C_q_ value^b^	Average C_q_ value
rPR8wt	6.00×10^7^	1.15×10^8^	19.06	19.16
	1.55×10^8^		19.37	
	1.30×10^8^		19.08	
rPR8 M1 N87S	5.00×10^7^	7.60×10^7^	19.23	19.32
	5.00×10^7^		19.42	
	1.30×10^8^		19.32	
rPR8 M1 R101G	4.00×10^7^	7.80×10^7^	18.86	18.94
	6.50×10^7^		18.92	
	1.30×10^8^		19.04	

a rPR8wt to rPR8 M1 N87S comparison (p = 0.38), rPR8wt to rPR8 M1 R101G comparison (p = 0.40). A two-tailed Student *t*-test was used to assess significance.

b rPR8wt to rPR8 M1 N87S comparison (p = 0.23), rPR8wt to rPR8 M1 R101G comparison (p = 0.10). To assess significance, a two-tailed Student *t*-test was applied to values of 2^(−Cq)^.

### Red blood cell elution assay

Each virus was standardized to a concentration of 128 HAU. Duplicate HA assays were then set up in parallel using 1∶2 serially diluted viruses and allowed to develop at 4°C. One set of plates was then transferred to 37°C (t = 0 hours) to trigger neuraminidase activity, while the second set of plates was left at 4°C to act as a negative control. Red blood cells were monitored for elution (visible as the formation of a red blood cell pellet at the bottom of the well) at the following time points: 1, 2, 3, 4, 6, and 8 hours.

### MUNANA neuraminidase activity assay

Neuraminidase activity assays using the soluble substrate methylumbelliferyl N-acetylneuraminic acid (MUNANA) were performed as previously described by Campbell et al. [20]. Virus was diluted to 5×10^5^ PFU/ml and 80 µl was added to each well of a black 96-well plate (CoStar). A sample of each diluted virus preparation was retained for quantification of viral RNA therein by RT-qPCR. Concentrations of MUNANA substrate ranging from 1.17 µM to 150 µM were used. When cleaved by the viral NA, MUNANA produces a fluorescent product. Fluorescence was quantified using a Biotek Synergy H1 plate reader every minute over the course of an hour. Fluorescence curves were then fitted to the Michaelis-Menton equation to determine values of V_max_ (maximal enzyme velocity) and K_m_ (the Michaelis constant, the substrate concentration at which the reaction rate is half of V_max_). Each experiment included triplicate samples of each virus.

### Virus concentration for Western blot analysis

Each virus was purified from allantoic fluid collected from 9–11 day embryonated chicken eggs infected with 250 PFU of virus. Allantoic fluid was spun at 3,000 rpm for 10 minutes at 4°C in a Sorvall tabletop centrifuge after which the supernatant was transferred to ultracentrifuge tubes (Beckman Coulter). Samples were spun in an SW32 rotor at 10,000 rpm for 30 minutes at 4°C and supernatant was then transferred to a fresh tube where a 5-ml 30% sucrose cushion was added. Samples were spun in an SW32 rotor at 25,000 rpm for 1.5 hours at 4°C. All supernatant was removed and 100 µl of PBS was added to the virus pellet and allowed to resuspend at 4°C overnight.

### NP normalization and Western blot

Concentrated virus samples were denatured by boiling for 10 minutes and treated with PNGaseF (New England Biolabs) for 1 hour at 37°C to allow for deglycosylation. The amount of NP in each virus sample was quantified by polyacrylamide gel electrophoresis followed by Coomassie staining (GelCode Blue – Thermo Scientific) and analysis with Image Lab software (Bio-Rad). The volume of each sample used for western blotting was then normalized based on NP content. Samples were loaded on a 4–15% SDS gradient gel (Bio-Rad Mini Protean) and electrophoresed at 130 V for 1 hour and 5 minutes. Protein was transferred (semi-dry) onto nitrocellulose membrane for 1 hour at 100 mA and blocking was performed overnight. NA was detected using a goat anti-NA primary antibody (BEI NR-9598) and a donkey anti-goat alexa 647-conjugated secondary antibody. NP was detected using a rabbit anti-NP primary antibody (a kind gift of Peter Palese) and a donkey anti-rabbit alexa 488-conjugated secondary antibody. Band intensity was quantified using Image Lab software (Bio-Rad).

### Red blood cell-based avidity assay

The red blood cell-based avidity assay was performed similarly to those described in [21]. Briefly, a 1.3% solution of red blood cells in PBS was treated with a series of dilutions of *C. perfringens* neuraminidase (Sigma) for 30 minutes at 37°C. Neuraminidase concentrations incremented by 5 mU/ml for the PR8-based viruses and 10 mU/ml for the H3 and H7 subtype viruses. Treated red blood cells were then added to virus at a standardized concentration of 8 HAU in a v-bottom, 96-well plate. Hemagglutination was assessed after 2 hours at 4°C. To rule out the activity of the viral NA in interpreting results, virus was diluted in PBS containing oseltamivir carboxylate (GS4071) and the assay was allowed to develop at 4°C.

### Trypsin-heat-periodate treatment

In order to remove nonspecific inhibitors of hemagglutination, serum treatment was performed as outlined in [22]. Briefly, serum was treated with L -1-Tosylamide-2-phenylethyl chloromethyl ketone (TPCK) trypsin for 30 minutes at 56°C. After cooling to room temperature, serum was then treated with 0.011 M metapotassium periodate (KIO_4_) for 15 minutes at room temperature. After KIO_4_ treatment, serum was treated with 1% glycerol in PBS for 15 minutes at room temperature after which an 85% PBS solution was added to reach a final serum dilution of 1∶10.

### Hemagglutination inhibition (HI) assay

Trypsin-heat-periodate-treated anti-PR8 guinea pig serum was diluted in PBS either 1∶2 or 1∶1.5 across a v-bottom, 96-well plate. Each virus was standardized to a concentration of 8 HAU and was then added to the diluted serum. Serum and virus were incubated together at 4°C for 30 minutes after which 0.5% red blood cells in PBS were added to each well. The assay was allowed to develop at 4°C. Naïve guinea pig serum was used as a negative control. The HI titers reported reflect HI activity above background levels.

### Plaque reduction assay

Each virus was diluted to approximately 250 PFU. Trypsin-heat-periodate treated anti-PR8 guinea pig serum was serially diluted 1∶80, 1∶160, 1∶320, and 1∶640 in PBS. Control serum obtained from a naïve guinea pig was also used. Virus was added to the diluted serum and incubated for 30 minutes at 37°C. The infectious titer of each serum/virus sample then quantified by plaque assay in MDCK cells. This assay was performed in triplicate.

### Thermostability assay

Each virus was diluted to approximately 1×10^6^ PFU. Fifteen 120 µl aliquots of each virus were incubated at 50°C and three aliquots of each virus were removed at 0, 15, 30, 60, and 120 minutes. Titers for each sample were quantified via plaque assay on MDCK cells.

## Results

### Equivalent HAU of the rPR8wt, rPR8 M1 N87S, and rPR8 M1 R101G viruses do not differ in infectivity or RNA copy number

Each of the functional assays that we applied to our spherical and filamentous PR8 viruses required normalization of the input of each virus. Ideally, this normalization would be achieved by counting virus particles in transmission electron micrographs, but this approach was not practical given that the preparations used were relatively dilute and concentration by centrifugation can alter virus morphology. We therefore performed the following experiment to determine the relationships between the hemagglutination-based titer, infectious (PFU) titer and RNA copy number for each virus. rPR8wt, N87S, and R101G viruses were diluted to 128 HAU and HA titers were confirmed. Diluted virus samples were then titrated in triplicate by plaque assay. The mutant viruses showed lower PFU titers compared to rPR8wt, but the differences were not statistically significant (Table 1). RNA was extracted from 160 µl of each diluted virus and quantified by reverse transcription followed by quantitative PCR. C_q_ values obtained were consistent across all three viruses (Table 1). Thus, for all three viruses, the HAU to PFU and HAU to RNA copy number ratios were comparable. To assess the precision and consistency of the hemagglutination assay, we furthermore evaluated the PFU titers and relative RNA copy numbers of three 128 HAU samples of rPR8wt virus that had been obtained through independent dilution series. The average PFU titers obtained ranged from 1.66 to 1.78×10^8^ PFU/ml (n = 3 per 128 HAU sample). The average C_q_ values were also very similar, ranging from 18.64 to 18.85. Taken together, these results indicate that similar results would be expected following normalization of rPR8wt, N87S, and R101G viruses by PFU, HAU or RNA copy number.

### The rPR8 M1 N87S and rPR8 M1 R101G filamentous mutants have higher neuraminidase activity than rPR8wt virus

**Figure 1:**
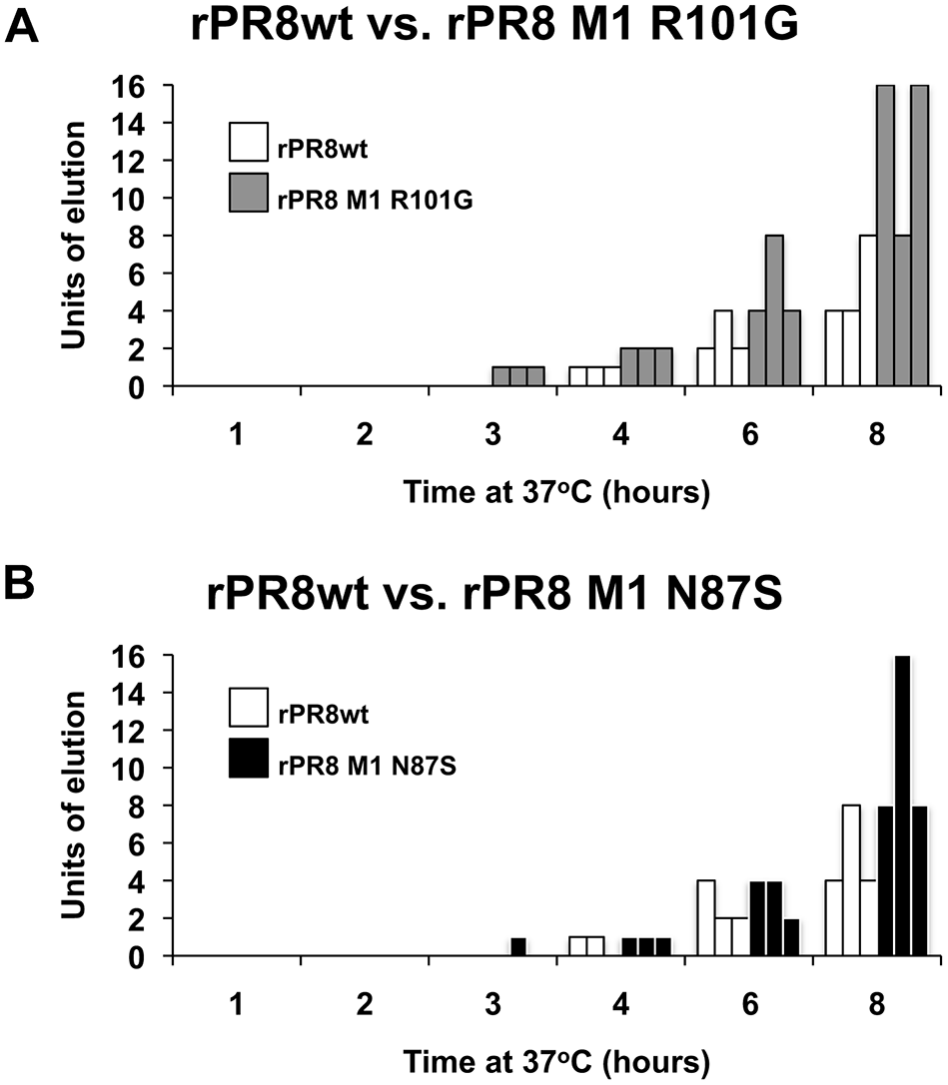
rPR8 M1 R101G and rPR8 M1 N87S viruses elute red blood cells at a faster rate than rPR8wt virus. HA assays were set up using virus diluted to a concentration of 128 HAU. After the assays had developed, plates were transferred to 37°C to allow for red blood cell elution by the viral neuraminidase. A second set of plates remained at 4°C where no elution occurred (not shown). The results of three independent experiments are shown, with each experiment represented by a separate bar. Within each experiment, viruses were analyzed in triplicate (standard deviation for each virus = 0). “Units of elution” is defined as the reciprocal of the highest virus dilution showing elution. A) Elution of rPR8 M1 R101G virus is compared to that of rPR8wt virus. B) Elution of rPR8 M1 N87S virus is compared to that of rPR8wt virus.

The NA activities of the spherical and filament-producing viruses were assessed by comparing rPR8wt, N87S, and R101G viruses in a red blood cell elution assay. To test our hypothesis that filamentous and spherical virions differ at the level of the whole virus particle, due to differing surface areas, we aimed to evaluate NA activity per virion rather than per NA protein. We therefore normalized virus input by hemagglutination titer rather than protein levels. Briefly, HA assays were set up in parallel using a standardized amount of virus (128 HAU) and allowed to develop at 4°C. At that point, one set of plates was transferred to 37°C. At this temperature, the viral NA is active and begins cleaving the sialic acids on the surface of the red blood cells, causing them to drop to the bottom of the well (elution). We monitored the plates for elution over the course of 8 hours. Progressive elution was observed at 37°C, while no elution was seen over the same time period at 4°C. We found that, when incubated with the R101G mutant virus, red blood cells eluted at a faster rate compared to those incubated with rPR8wt (Figure 1A). The N87S mutant had a less marked phenotype than the R101G mutant, but also eluted red blood cells at a faster rate than the rPR8wt – particularly at the later time points (Figure 1B).

### The rPR8 M1 N87S and rPR8 M1 R101G filamentous mutants have higher neuraminidase activity in the MUNANA assay than rPR8wt virus

To confirm that the differing elution phenotypes observed were due to differing NA activities, we compared the spherical and filamentous rPR8 viruses using a MUNANA-based assay. MUNANA (methylumbelliferyl N-acetylneuraminic acid) is a soluble substrate that produces a fluorescent product when cleaved by the viral NA. For this assay, viruses were standardized to equivalent PFU and RNA titers and concentrations of MUNANA substrate ranging from 1.17 µM to 150 µM were used. Levels of fluorescence were measured every minute over a sixty-minute period. The resulting fluorescence curves were then fitted to the Michaelis-Menton equation for calculation of K_m_ and V_max_ values associated with each virus. It is important to note that, in line with our aim of evaluating NA enzyme kinetics per virion, NA protein levels contained within each virus sample were not normalized for this assay.

**Figure 2:**
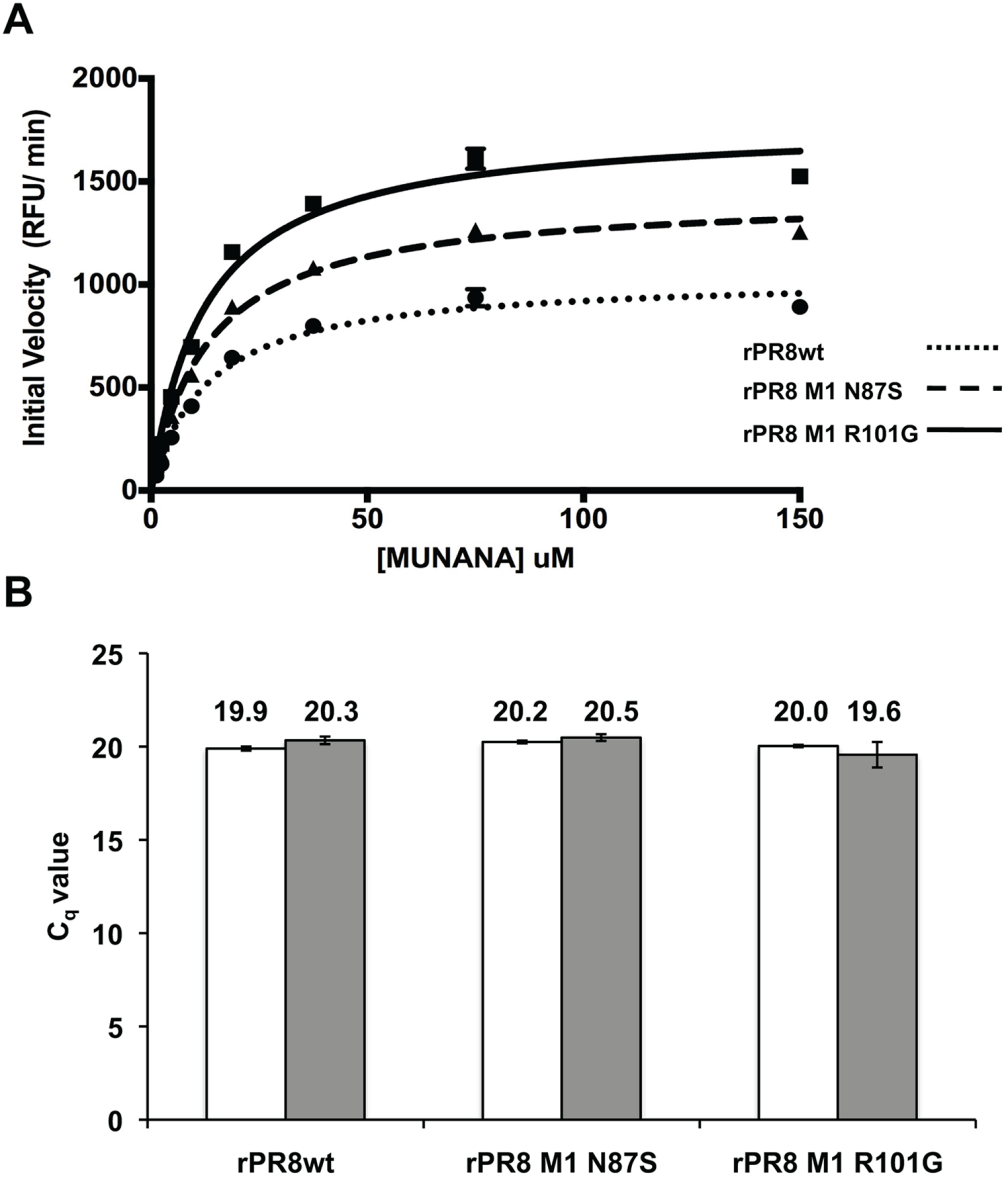
rPR8 M1 R101G and rPR8 M1 N87S viruses have higher neuraminidase activity than rPR8wt virus. A) Neuraminidase enzyme kinetics. Virus input was standardized to 5×10^5^ PFU and concentrations of MUNANA substrate ranging from 1.17 µM to 150 µM were used. Fluorescence generated at each time point (every minute over the course of 1 hour) was detected using a Biotek Synergy H1 plate reader. The resulting fluorescence curves were then fitted to the Michaelis-Menton equation. B) That equivalent amounts of each virus were used in the MUNANA assay was confirmed by RT-qPCR for the viral NP segment. The arithmetic mean (n = 3) and standard deviation of 2^(−Cq)^ values were calculated and then converted back to a C_q_ scale by taking the log_2_. Two biological replicates of each virus are included in white and grey bars; each biological replicate comprised three technical replicates.

The results obtained correlated well with those observed from the red blood cell elution assay (Figure 2). We confirmed that equivalent amounts of each virus were assayed by performing RT-qPCR on viral RNA extracted from the same diluted virus preparations employed in the MUNANA assay (Figure 2B). The R101G mutant virus displayed the highest V_max_, followed by the N87S mutant. rPR8wt had the lowest V_max_ (Table 2). The K_m_ for all three viruses was found to be consistent, as expected considering the NA protein is the same for all three viruses (Table 2). Based on the results of both the elution assay and the MUNANA assay, we concluded that the filament-producing M1 mutant viruses had a higher neuraminidase activity per virion than the spherical wild-type virus.

**Table 2:** rPR8 M1 N87S and rPR8 M1 R101G viruses have a higher neuraminidase activity than rPR8 wt virus in a MUNANA-based assay.

Virus	V_max_	V_max_ 95% confidence interval	K_m_	K_m_ 95% confidence interval
rPR8wt	1040	978–1101	13.02	10.41–15.63
rPR8 M1 N87S	1434	1370–1497	13.15	11.17–15.14
rPR8 M1 R101G	1785	1669–1901	15.52	10.60–20.44

### When standardized to NP protein levels, NA and M1 protein incorporation among rPR8wt, rPR8 M1 N87S, and rPR8 M1 R101G is similar

**Figure 3:**
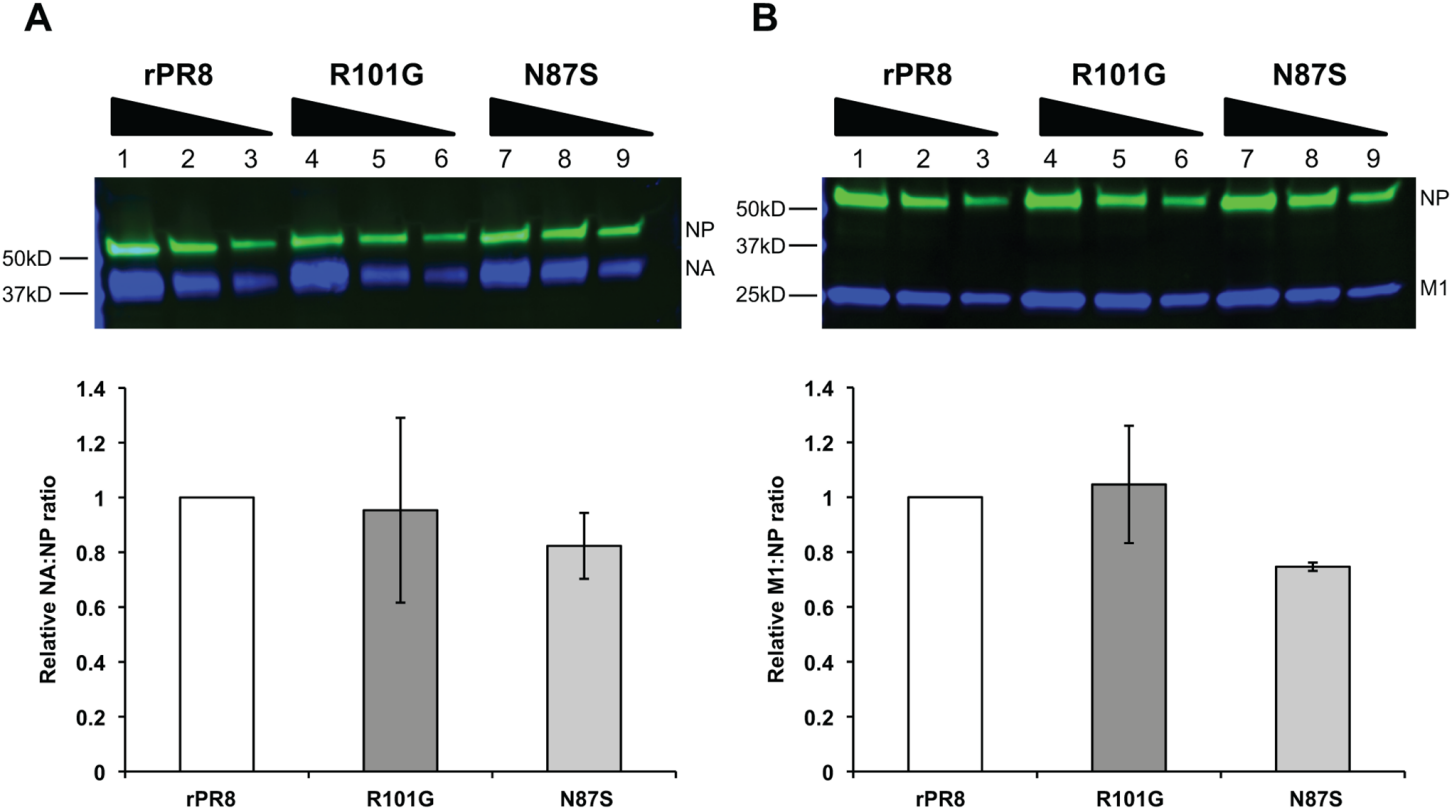
NA and M1 protein levels between rPR8wt, rPR8 M1 N87S, and rPR8 M1 R101G are similar when normalized to NP protein levels. Virus samples were concentrated via ultracentrifugation through a 30% sucrose cushion and resuspended in PBS. Samples were then deglycosylated and denatured, after which NP protein levels were standardized via Coomassie. For the Western blot, protein was detected using primary antibodies specific for NA, M1, and NP and fluorophore-conjugated secondary antibodies. All bands were quantified using Image Lab software (Bio-Rad). Error bars represent standard deviation.

Because filamentous virions can be much greater in size than spherical virions, a logical explanation for our NA activity results is that filamentous virions have more NA adorning their surface. Indeed, the consistent K_m_ values across all three viruses obtained from the MUNANA assay suggest that the intrinsic NA activity is unaffected. To test this hypothesis, we performed Western blots on concentrated virus preparations of rPR8wt, N87S, and R101G, and probed for NP, NA, and M1 proteins. The fluorescence intensity of bands was quantified using a Bio-Rad Chemidoc imager. NP and M1 were included as controls: due to its association with the viral genome, NP levels would be expected to be constant among viruses of differing morphology, while M1 levels would be expected to increase with surface area. For a given amount of NP, the filamentous mutant viruses did not show increased quantities of NA or M1 proteins in virions (Figure 3A and 3B). Similar results were obtained when egg-grown virus stocks were analyzed directly (rather than concentrating them first), and when inputs were normalized by viral RNA content, rather than normalization to NP (data not shown). Based on our inability to detect increases in M1:NP ratios for the filament-producing viruses relative to rPR8wt, we concluded that the Western blot assay used was not sufficiently sensitive to detect differences (or lack thereof) in NA or M1 incorporation. The preponderance of spherical viruses present in the N87S and R101G virus stocks most likely obscures any differences in protein content between spherical and filamentous particles.

### No difference in binding avidity is observed between rPR8wt, rPR8 M1 N87S, and rPR8 M1 R101G

**Figure 4:**
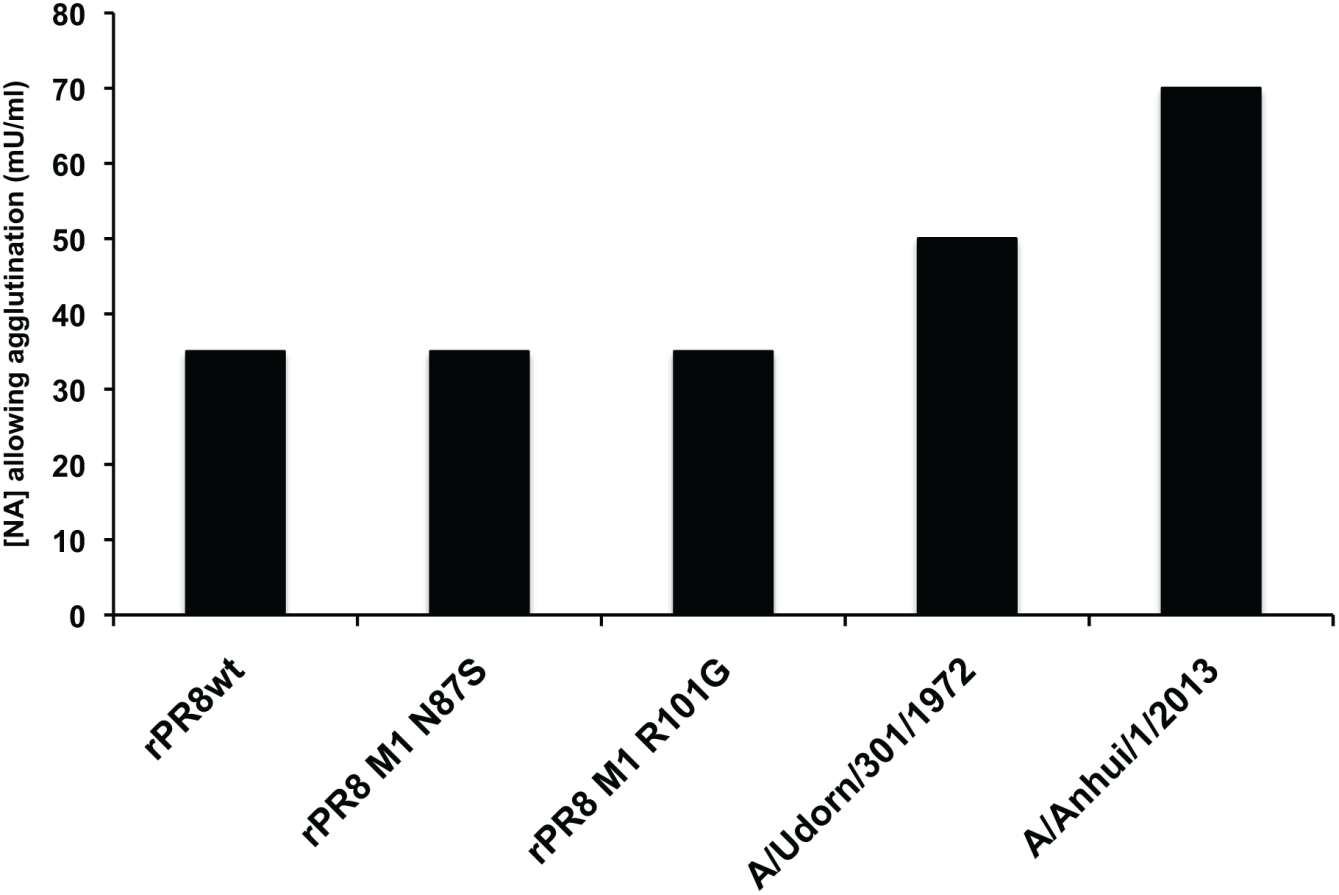
rPR8wt, rPR8 M1 N87S, and rPR8 M1 R101G viruses have the same red blood cell binding avidity. Chicken red blood cells were treated with a series of dilutions of *C. perfringens* neuraminidase. Treated red blood cells were then added to a standardized amount of each virus (8 HAU) and allowed to develop at 4°C. The assay was run in triplicate (standard deviation for each virus = 0). The highest concentration of neuraminidase that still allowed agglutination by each virus is plotted.

After assessing the NA activity between spherical and filamentous viruses, we compared the function of the viral HA in a red blood cell-based avidity assay. We treated chicken red blood cells with a series of dilutions of *C. perfringens* neuraminidase. This treatment removes alpha 2,3-, alpha 2,6- and alpha 2,8-linked sialic acids. Thus, red blood cells treated with higher concentrations of neuraminidase had fewer sialic acids on their surface than those treated with lower concentrations of neuraminidase. We then added a standardized amount of virus (8 HAU) to the treated red blood cells and allowed agglutination to occur. We found that the neuraminidase concentration that prevented agglutination was the same for the rPR8wt, N87S, and R101G viruses, indicating that red blood cell binding avidity is not affected by the changes in morphology seen with these viruses (Figure 4). Additionally, to validate that the assay was sufficiently sensitive to detect differences in red blood cell binding avidity, we compared avidity of the rPR8-based viruses to those of viruses with differing HA types (specifically, H3 and H7). We found that A/Udorn/301/1972 (H3N2) and BPL-inactivated A/Anhui/1/2013 (H7N9) virus had higher red blood cell binding avidities than the PR8-based viruses. From these results, we concluded that the changes in virion morphology mediated by the N87S and R101G mutations do not affect the binding avidity of rPR8 virus.

### No difference in hemagglutination inhibition and little difference in plaque reduction was observed among rPR8wt, rPR8 M1 N87S, and rPR8 M1 R101G

Next, we compared hemagglutination inhibition (HI) between rPR8wt, N87S, and R101G viruses, reasoning that, due to their increased size, filaments may be more difficult to neutralize than spheres. Using 1∶2 dilutions of trypsin-heat-periodate treated anti-PR8 guinea pig immune serum and virus standardized to 8 HAU, we found no difference in HI among rPR8wt, N87S, and R101G (Table 3). Since we were working with populations of mixed morphology, which may make differences between filaments and spheres difficult to detect, we sought to improve the sensitivity of the assay by using a series of 1∶1.5 serum dilutions. Similar to the assays utilizing 1∶2 dilutions, we observed little difference in HI between the spherical wild-type and filamentous mutants (Table 3).

**Table 3:** There are no differences in hemagglutination inhibition among rPR8wt, rPR8 M1 N87S, rPR8 M1 R101G viruses.

	1∶2 serum dilutions			1∶1.5 serum dilutions		
	HI titer^a^			HI titer^a^		
Virus	A	B	C	A	B	C
rPR8wt	160	160	320	256	256	384
rPR8 M1 N87S	160	160	160	256	256	384
rPR8 M1 R101G	160	160	320	171	256	384

a The reciprocal of the highest dilution of serum that prevented hemagglutination is shown for three replicates (A, B and C).

**Figure 5:**
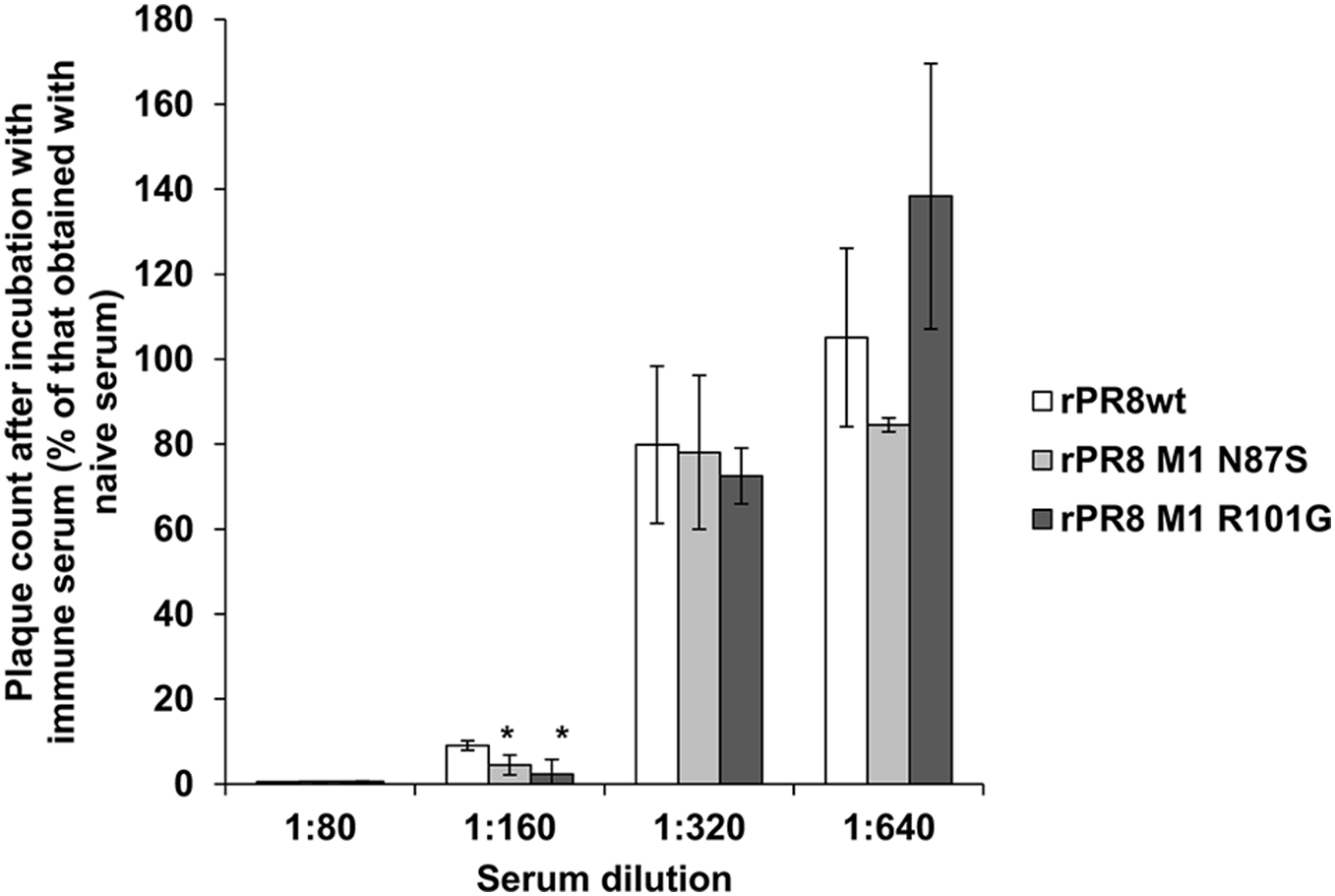
There is little difference in plaque reduction among rPR8wt, rPR8 M1 N87S, and rPR8 M1 R101G viruses. Each virus was diluted to approximately 250 PFU. Trypsin-heat-periodate treated serum was diluted 1∶80, 1∶160, 1∶320, and 1∶640 in PBS. Virus was added to serum dilutions and incubated for 30 minutes at 37°C. Virus titer for each serum/virus sample was then quantified by plaque assay on MDCK cells. The number of plaques obtained following incubation with immune serum is plotted as a percentage of the plaques obtained following incubation with naïve serum. The mean of three replicates is plotted, and error bars indicate standard deviation. *p<0.05 compared to rPR8wt virus. The limit of detection for the plaque assays was 5 PFU/ml.

To substantiate the relationship between particle morphology and sensitivity of virus to immune serum, we also performed a plaque reduction assay. Briefly, trypsin-heat-periodate treated anti-PR8 guinea pig serum was diluted to 1∶80, 1∶160, 1∶320, or 1∶640 in PBS. The same dilutions of a naïve guinea pig serum were used as controls. Virus was diluted to approximately 250 PFU and incubated with the diluted serum for 30 minutes at 37°C. Following the incubation period, virus titer was determined in triplicate for each serum/virus sample by plaque assay on MDCK cells. Plaque numbers were compared to those obtained when virus was incubated with the control serum. All three viruses showed reductions in titer with immune serum diluted 1∶80 and 1∶160, but not with immune serum diluted 1∶320 or 1∶640 (Figure 5). The extent of reduction seen with the wt vs. mutant viruses at the 1∶160 dilution was found to be significantly different for both mutants (p<0.05, t-test) and suggested that the two filamentous viruses are slightly more sensitive to antibody neutralization than is the spherical PR8wt strain (Figure 5). We interpret these results with caution, however, since the number of plaques remaining after incubation with 1∶160 diluted immune serum was near the limit of detection. Overall, the results from the HI assays and the plaque reduction assay indicate that the greater surface area of filamentous compared to a spherical viruses does not confer resistance to antibody neutralization.

### No difference in virion stability is observed between rPR8wt, rPR8 M1 N87S, and rPR8 M1 R101G

**Figure 6:**
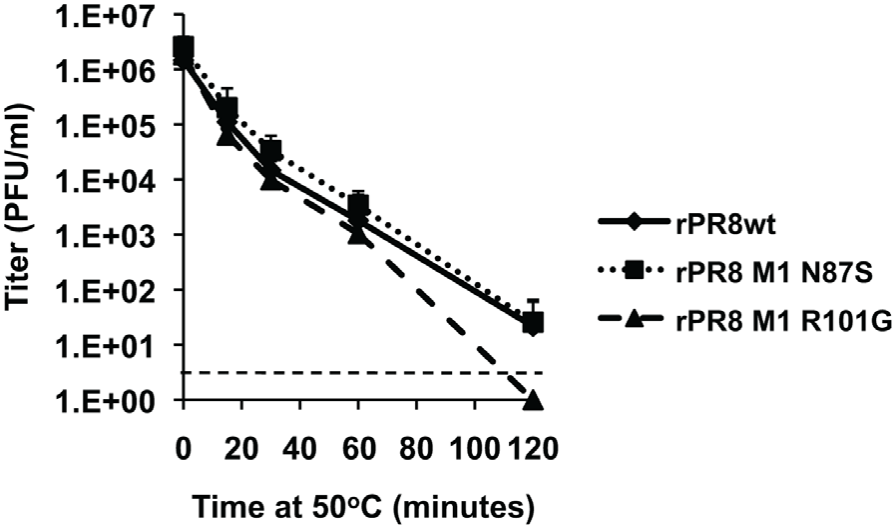
No difference in virion stability at an elevated temperature was observed between rPR8wt, rPR8 M1 N87S, and rPR8 M1 R101G viruses. Each virus was diluted to 1×10^6^ PFU and incubated in triplicate at 50°C for one of the following lengths of time: 0, 15, 30, 60, and 120 min. Results shown are the average of four separate assays performed in triplicate (thus n = 12). The titers of each mutant virus were compared to that of the wt virus at each time point using Student’s *t* test. All p values were >0.05 except that obtained for wt vs. N87S viruses at the 0 h time point (p = 0.0094). The dotted line indicates the limit of detection.

Due to potential differences in the structure of the matrix layer [23], we hypothesized that filamentous and spherical viruses might differ in their sensitivity to environmental stresses, such as fluctuations in temperature. To test this hypothesis, we evaluated virion stability at high temperatures. Each virus was diluted to a concentration of 1×10^6^ PFU and incubated at 50°C for one of the following lengths of time: 0, 15, 30, 60, or 120 minutes. Following heat exposure, the titer of each sample was quantified in triplicate via plaque assay in MDCK cells. All three viruses had similar infectious titers remaining at each time point, indicating that the observed changes in virion morphology do not affect virion stability at elevated temperatures (Figure 6).

## Discussion

The fact that the filamentous morphology of IAV is maintained in nature but not in the laboratory suggests that filaments have a functional significance within the infected host. Due to the greater surface area of filaments relative to spheres, we hypothesized that functional differences between the two morphologies may lie with the HA and NA surface glycoproteins. We therefore focused our study on the HA and NA functions of strains with differing morphological phenotypes. We took advantage of two M1 point mutants selected during serial adaptation of rPR8 virus to an animal host [5]. By measuring particles in electron micrographs, the R101G mutant was previously shown to comprise 41% filamentous particles, while the N87S had 16% filaments and the rPR8wt virus had 4% filaments [5].

Our approach, focused on the surface of the virion, assumes that the internal components of spherical and filamentous particles are similar. Our results indicating comparable infectivity and RNA content per HAU for spherical and filament-producing strains supports this assumption. Similar results were also reported by Roberts et al. for the A/Udorn/301/1972 (H3N2) strain [10]. Nevertheless, the literature contains conflicting reports on the genomic content of filaments versus spheres. Early studies suggested that filamentous virions could be polyploid (containing more than one copy of the genome) or contain more RNA than their spherical counterparts [24], [25]. In contrast, a recent cryo-electron tomography study has shown that many longer filaments produced by A/Udorn/301/1972 (H3N2) virus lack RNPs [26]. Lastly, sectioning TEM and cryo-electron tomography studies have shown that filamentous virions contain a single copy of the viral genome located at the apical tip of the budding virion [23], [27]. These apparently contradictory results can be partially reconciled by noting that the absence of genomes from filamentous particles appears to apply mainly to very long filaments [26]. In some cases, IAV strain specific differences in the properties of filaments may also play a role.

Our observations through two independent functional assays show that the two filament-producing rPR8 mutants have higher NA activities than the spherical rPR8wt virus. Replacement of the PR8 M segment with that of the filamentous 2009 pandemic strain A/Netherlands/602/2009 (H1N1) was also shown to increase both filament production and NA activity compared to the rPR8wt virus [20]. Now we show that significant increases in NA activity can be conferred through a single point mutation that changes virion morphology, thereby strengthening the causal link between morphology and NA activity. We predict that the increased NA activities associated with filament-containing virus preparations are due to greater numbers of NA proteins adorning the surface of filaments compared to spheres. We were not able to test this prediction robustly, however, due to limitations in the sensitivity of our Western blot assay. An alternative mechanism by which morphology could impact NA activity relates to the distribution of NA molecules on the virion surface. If filaments and spheres differ in terms of the positioning of NA on the particle, increased neuraminidase activity could be due to a cooperative effect mediated by greater NA protein clustering on filamentous virions. Consistent with this idea, clustering of NA at the tip of the virus particle proximal to the cell membrane has been reported [23], [28], [29]. Such an arrangement was suggested to promote destruction of host cell receptors as the virus is budding.

Contrary to what was observed for NA activity, we found no difference in binding avidity to red blood cells between the spherical and filamentous rPR8 viruses. Similarly, we did not observe marked differences in HI titer or in plaque reduction between spherical and filamentous viruses. These results suggest that the mechanism by which NA activity is increased for filament-producing viruses does not apply to HA. For example, if incorporation of both glycoproteins increases with filament size, then our data would suggest that avidity does not increase linearly with the valency of the virus particle. Lastly, we did not observe a difference in thermostability between spherical and filamentous viruses. Thus our data suggest that, at least in the PR8 background and in a guinea pig host, the selective advantage of a filamentous morphology lies with increased NA activity.

Enhanced NA activity could be advantageous to the virus by promoting release from infected cells and/or spread within the respiratory tract to new target cells [30]. Indeed, Roberts et al. suggested that increased amounts of NA protein per virion could aid movement through the mucus lining the airway [31]. Additionally, increased NA activity was shown to improve transmission in guinea pigs [20], [32]. Importantly, the M segment has been shown to affect virus transmission as well [20], [33]. Whether this effect on transmission is directly mediated by viral morphology has not yet been established. However, the fact that a single point mutation in the M1 matrix protein can both significantly alter virion morphology and confer increased NA activity suggests a mechanism by which the M segment may affect transmission. We know from previous work that the rPR8 M1 N87S mutant virus, which has both a significantly more filamentous morphology as well as a higher NA activity than rPR8wt, is not transmissible by a contact route in the guinea pig transmission model [5]. It is likely that N87S and the other point mutations identified in M1 following serial passage need to be coupled with additional permissive mutations on the M segment and/or elsewhere in the genome to promote transmission [34].

In summary, we have shown that filament-producing viruses have a higher neuraminidase activity than their spherical counterparts. Other properties such as HA binding avidity, HI titer, and thermostability were unaffected by changes in virion morphology. The viruses used herein were single M1 point mutants generated on a PR8 background and produced significantly more filaments than rPR8wt. The fact that these point mutations, when introduced individually, confer both a filamentous morphology and increased NA activity further strengthens the idea that the selective advantage conferred by filamentous virions lies in their increased NA activity over spherical virions.
